# Supplementary material for: Q-dependent collective relaxation dynamics of glass-forming liquid Ca0.4K0.6(NO3)1.4 investigated by wide-angle neutron spin-echo
Source: Nat Commun. 2022 Apr 19;13:2092. doi: 10.1038/s41467-022-29778-4 (PMC9018732; doi:10.1038/s41467-022-29778-4)
Supplement: Supplementary file 1 — Supplementary Information [file 41467_2022_29778_MOESM1_ESM.pdf]

Supplementary Information for

Q-dependent Collective Relaxation Dynamics of Glass-Forming Liquid $Ca_{0.4}K_{0.6}(NO_3)_{1.4}$ Investigated by Wide-Angle Neutron Spin-Echo

Peng Luo, Yanqin Zhai, Peter Falus, Victoria García Sakai, Monika Hartl, Maiko Kofu, Kenji Nakajima, Antonio Faraone, Y Z

The PDF file includes:

Supplementary Table1 Neutron scattering cross sections

Supplementary Figure1 DSC measurement for CKN

Supplementary Figure 2 Polarized diffraction data of CKN

Supplementary Figure 3 ISF measured on the NGA-NSE spectrometer

Supplementary Figure 4 Comparison of $\tau_{slow}/\tau_{slow}(Q = 1.86$ Å$^{-1})$ with $S(Q)/Q^2$

Supplementary Figure 5 The stretching exponent β as a function of temperature

Supplementary Figure 6 The relaxation time τ_{slow} as a function of 1000/T

Supplementary Figure 7 Macroscopic relaxation time of CKN

Supplementary Figure 8 Comparison of VFT fit by fixing T_0 or D and by fitting both

Supplementary Figure 9 The average relaxation time $\langle\tau_{slow}\rangle$ as a function of 1000/T

Supplementary Table 1. Neutron scattering cross sections (σ, σ_{coh} coherent scattering, σ_{inc} incoherent scattering, 1 barn = 10^{-28} m^2) for different elements present in CKN, adapted from https://www.nist.gov/ncnr/planning-your-experiment/scattering-length-periodic-table.

	σ_{coh} (barn)	σ_{inc} (barn)
N	11.01	0.5
O	4.232	0.0008
Ca	2.78	0.05
K	1.69	0.27

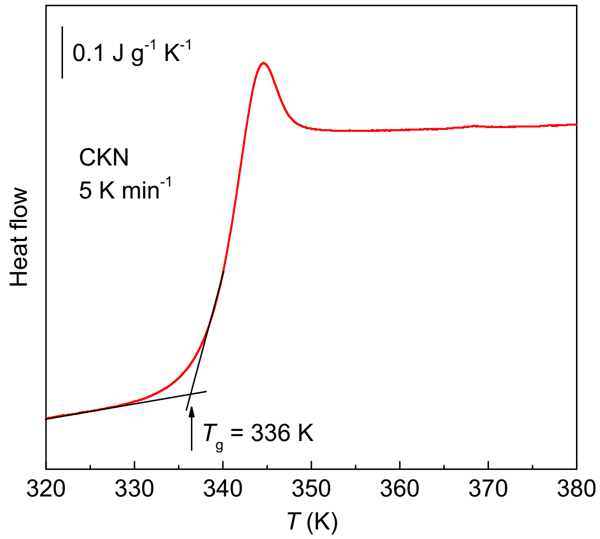

Supplementary Figure 1. Differential scanning calorimetry (DSC) for CKN glass performed on a Perkin-Elmer DSC 8000 in high-purity standard aluminum crucibles at a heating rate of 5 K min^{-1} under a constant flow of high-purity argon gas (20 ml min^{-1}). The onset glass transition temperature (T_g) as defined by the crossing of the black lines is 336 K. Source data are provided as a Source Data file.

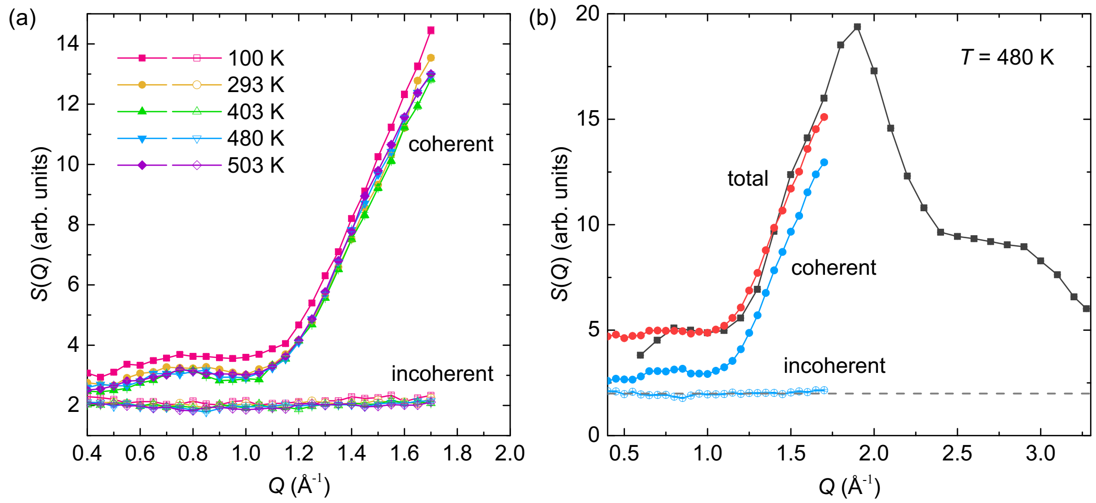

Supplementary Figure 2. Polarized diffraction data of CKN sample measured on the NGA-NSE spectrometer at the NCNR. (a) Coherent scattering (solid symbols) and incoherent scattering (open symbols) at different temperatures at 0.4 $Å^{-1} \leq Q \leq 1.7$ $Å^{-1}$. (b) Coherent scattering (solid blue circles) and incoherent scattering (open blue circles) at $T = 480$ K. The total scattering intensity (solid red circles) is the sum of the coherent and incoherent contributions to the scattering signal. The black squares are the total scattering intensity at $T = 480$ K measured on AMATERAS at J-PARC and normalized to overlap with the data obtained from polarized diffraction. Dash line is a guide for the eye. Source data are provided as a Source Data file.

From Supplementary Fig. 2(a), we can see that the incoherent scattering intensity remains independent of Q and temperature as expected. The normalized total scattering intensity measured on AMATERAS and the fact that the incoherent scattering intensity does not change with Q, allows for the estimation of the incoherent contribution at the higher Q's where polarized diffraction data is not available. As shown in Supplementary Fig. 2(b), for $T = 480$ K the total scattering intensity at $Q = 2.4$ $Å^{-1}$ is 9.64, while the incoherent scattering intensity is 2 being the same as at lower Q's. Note that in neutron spin echo measurements, polarized neutrons are used and so the dynamic signal is given by $I_{coh} - 1/3I_{inc}$. As a result, the incoherent contribution at $Q = 2.4$ $Å^{-1}$ is 1/3*2/(1/3*2+9.64) = 6.4%. As a comparison, we now look at $Q = 1$ $Å^{-1}$ where the

coherent scattering intensity is just 2.92 and thus the incoherent contribution is 1/3*2/(1/3*2+2.92) = 18.6%, which should be the largest value for CKN in the studied Q range of our spin echo experiments, i.e., from 0.8 Å^{-1} to 2.82 Å^{-1}. These calculations indicate that the incoherent contribution is very small in CKN in our studied Q range, and that the anomalous dynamic behavior observed for $Q > 2.4$ Å^{-1} cannot be a result of incoherent scattering. If it were, it will be more pronounced around $Q = 1$ Å^{-1} than at $Q > 2.4$ Å^{-1}. Furthermore, as shown in Fig. 6 in the main text, without any artificial adjustments to the data, the agreement between the intermediate scattering functions obtained from neutron spin echo and time-of-flight experiment, which involves contribution from all incoherent scattering, indicates that the incoherent contribution is negligible. We thus conclude that our observed dynamic features at $Q > 2.4$ Å^{-1} are not a result of the contribution from the incoherent component of the scattering.

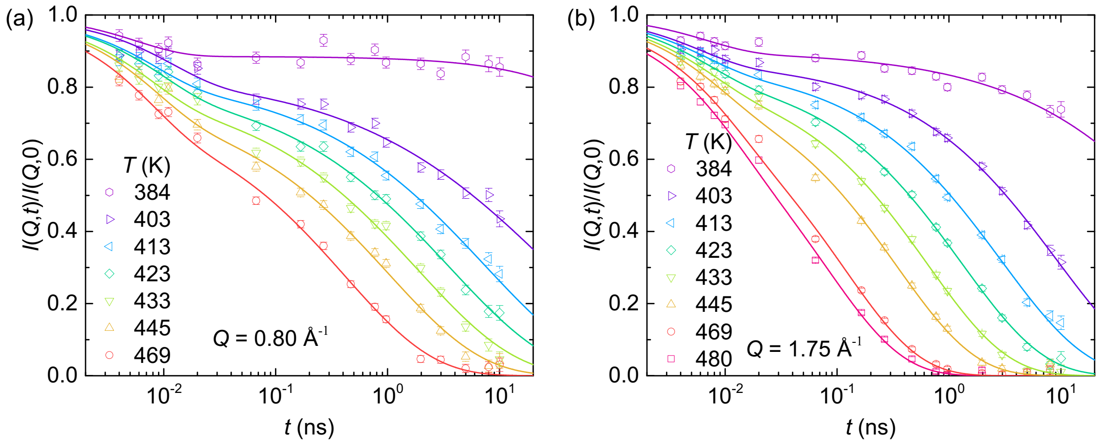

Supplementary Figure 3. (a) Intermediate scattering function of CKN as measured on the NGA-NSE spectrometer at NCNR for (a) Q = 0.80 Å^{-1} and (b) Q = 1.75 Å^{-1} at various temperatures. The solid lines represent fit to Eq. 1. Source data are provided as a Source Data file.

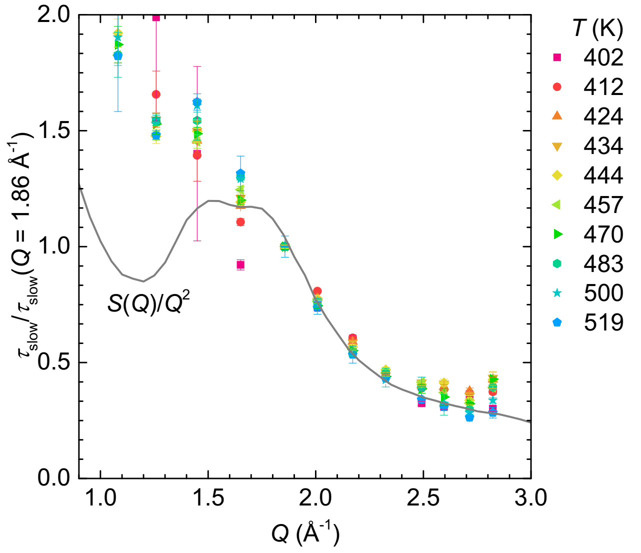

Supplementary Figure 4. Normalized relaxation time $\tau_{slow}/\tau_{slow}(Q = 1.86$ Å$^{-1})$ as a function of Q at various temperatures. The solid line represents $S(Q)/Q^2$, $S(Q)$ was measured at $T = 480$ K. The good agreement between the normalized relaxation time at 1.8 Å^{-1} < Q < 2.4 Å^{-1} is consistent with the very small change of D and T_0 from VFT fit as shown in Fig. 4(d), suggesting a similar temperature dependence of the relaxation time in this Q range.

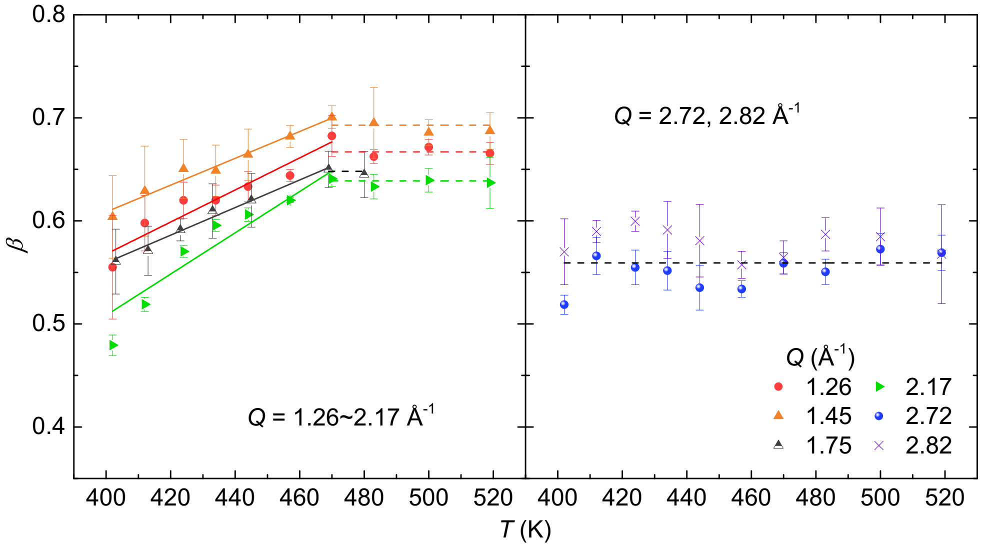

Supplementary Figure 5. The stretching exponent β as a function of temperature. Left panel: $Q = 1.26$ Å^{-1} to 2.17 Å^{-1}, right panel: $Q = 2.72$ Å^{-1} and 2.82 Å^{-1}. Solid lines are linear fit to the data in the temperature range of 402 K and 470 K. Dash lines are guide for the eye. At Q = 2.72 Å^{-1} and 2.82 Å^{-1}, the slope $\mathrm{d}\beta/\mathrm{d}T$ as shown in Fig. 4(d) are obtained from linear fits to the data in the entire temperature range between 402 K and 519 K. Source data are provided as a Source Data file.

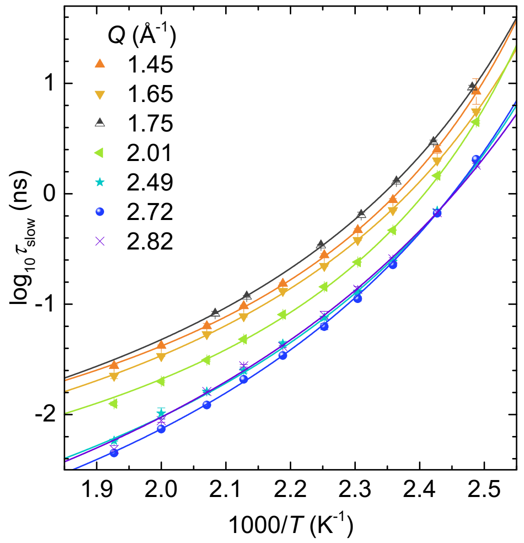

Supplementary Figure 6. The relaxation time τ_{slow} as a function of 1000/T with VFT fits (Eq. 2). Source data are provided as a Source Data file.

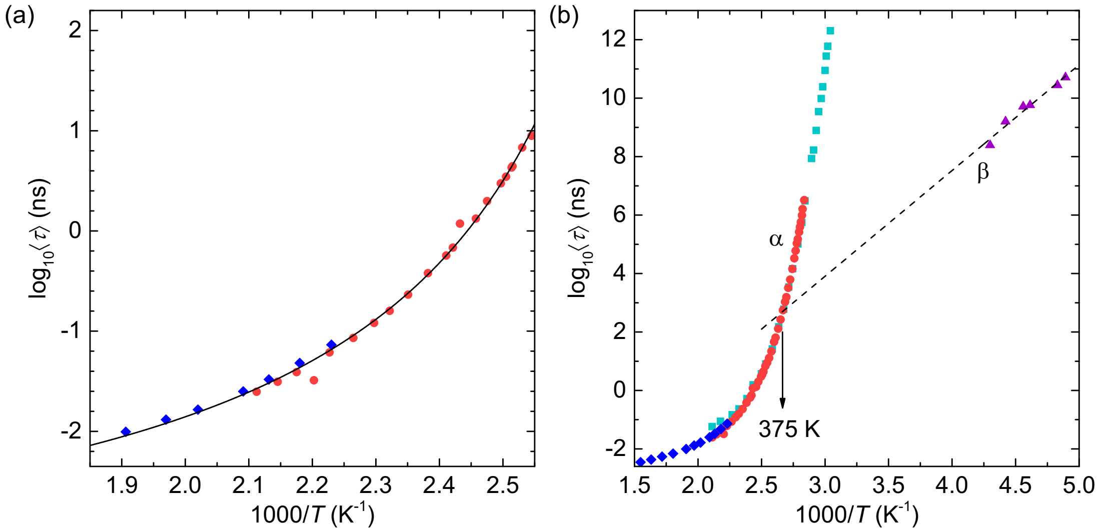

Supplementary Figure 7. Macroscopic relaxation time of CKN derived from the shear viscosity (α-relaxation) and the mechanical spectra (β-relaxation). (a) VFT fit (Eq. 2) to the macroscopic shear relaxation time $\langle\tau\rangle = \eta/G_\infty$ in the temperature range of the present study. $\langle\tau\rangle$ is calculated from the shear viscosity η (blue diamonds from Ref.[1], red circles from Ref.[2]) and the limiting high-frequency shear modulus G_∞ from Ref.[3]. (b) Extrapolation (dash line, Arrhenius fit) of the Johari-Goldstein β-relaxation detected by mechanical spectroscopy (purple triangles from Ref.[4]) suggests that the α to JG β crossover occurs at 375 K in CKN. The β-relaxation time are calculated by $1/2\pi f_{\max}$, where $f_{\max}$ is the peak frequency of the mechanical spectra. The blue diamonds and the red circles are the same data in (a), additional data of the shear relaxation time adapted from Ref.[5] are also shown as turquoise squares.

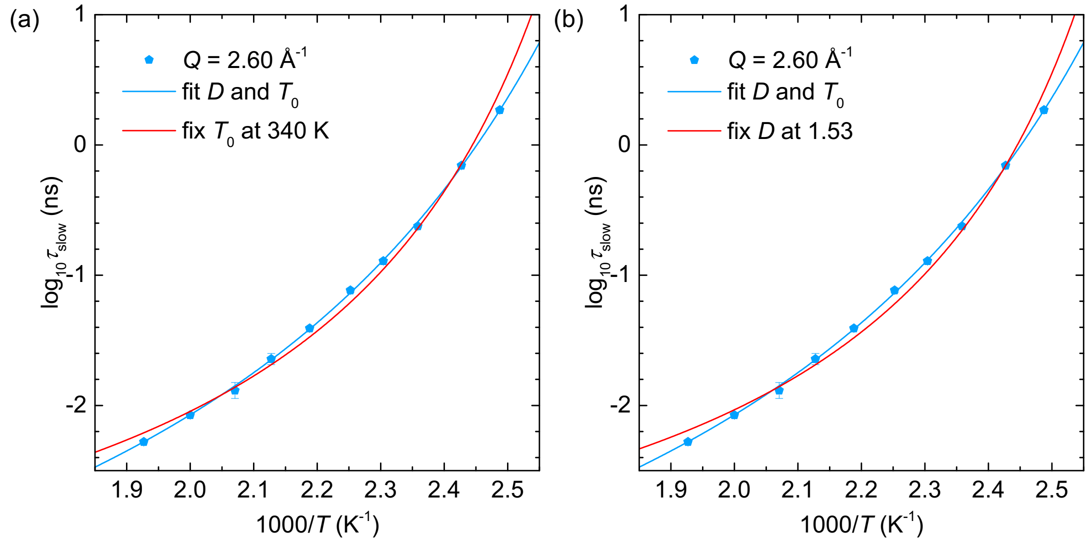

Supplementary Figure 8. Comparison of VFT fit (Eq. 2) to the relaxation time by fixing T_0 or D and by fitting both, taking the data at Q = 2.60 Å^{-1} as an example. The blue lines are fit with both T_0 and D free, while the red line in (a) is the best fit with T_0 fixed at 340 K and that in (b) with D fixed at 1.53, these values of D and T_0 are obtained from the macroscopic shear relaxation time shown in Supplementary Fig. 7. It is evident that fixing either T_0 or D prevents a satisfactory fit of the data.

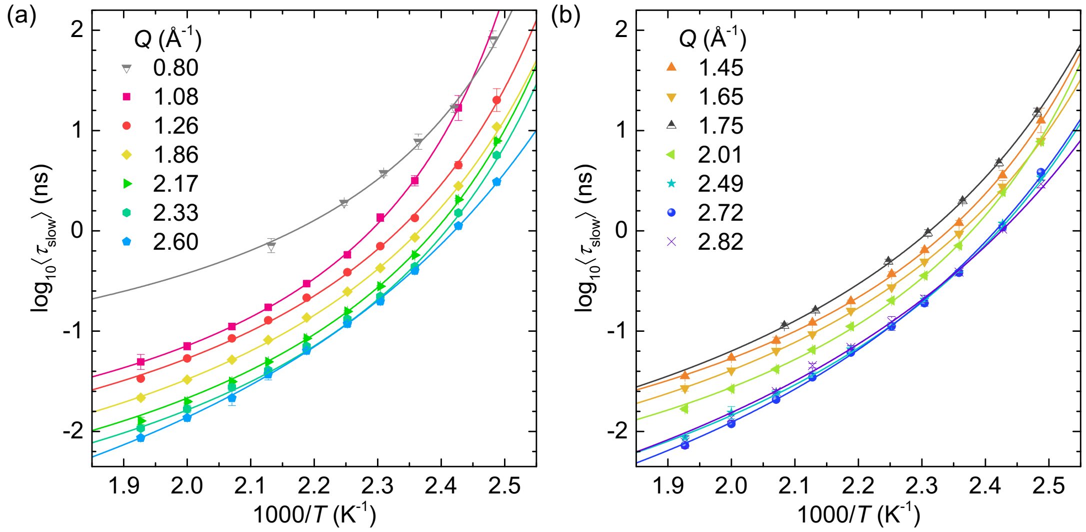

Supplementary Figure 9. The average relaxation time $\langle \tau_{\mathrm{slow}} \rangle$ as a function of $1000/T$ at various Q's, fitted with VFT law (Eq. 2). The data are shown in two panels, (a) and (b), to make it easier for the reader to discern the different curves. The half-filled triangles are obtained from the measurements on the NGA-NSE spectrometer at NCNR (Supplementary Fig. 3). Source data are provided as a Source Data file.

DISCLAIMER

Certain trade names and company products are identified to specify adequately the experimental procedure. In no case does such identification imply our recommendation or endorsement by the National Institute of Standards and Technology, nor does it imply that the products are necessarily the best for the purpose. Throughout the paper, error bars of the raw data represent one standard deviation, and error bars of the fitted parameters represent one standard error.

Supplementary References

1. Rhodes, E., Smith, W. E. & Ubbelohde, A. R. Melting and crystal structure: association in nitrate melts. *Proc. R. Soc. London. Ser. A* **285**, 263–274 (1965).
2. Weiler, R., Blaser, S. & Macedo, P. B. Viscosity of a vitreous potassium nitrate - calcium nitrate mixture. *J. Phys. Chem.* **73**, 4147–4151 (1969).

3. Torell, L. M. & Aronsson, R. Brillouin scattering study of elastic properties in a glass forming KNO 3 –Ca(NO 3) 2 mixture. *J. Chem. Phys.* **78**, 1121–1125 (1983).
4. Maï, C., Etienne, S., Perez, J. & Johari, G. P. Mechanical relaxation in an ionic glass. *Philos. Mag. B Phys. Condens. Matter; Stat. Mech. Electron. Opt. Magn. Prop.* **50**, 657–663 (1984).
5. Howell, F. S., Bose, R. A., Macedo, P. B. & Moynihan, C. T. Electrical relaxation in a glass-forming molten salt. *J. Phys. Chem.* **78**, 639–648 (1974).
